# Risk Factors Associated With Contrast-Induced Nephropathy after Primary Percutaneous Coronary Intervention

**DOI:** 10.7759/cureus.9721

**Published:** 2020-08-13

**Authors:** Dileep Kumar, Hussain Liaquat, Jawaid A Sial, Tahir Saghir, Rekha Kumari, Hitesh Kumar, Musa Karim, Kelash Rai, Reeta Bai

**Affiliations:** 1 Cardiology, National Institute of Cardiovascular Diseases, Karachi, PAK; 2 Medical Officer, Government of Sindh, Mithi, PAK; 3 Research, National Institute of Cardiovascular Diseases, Karachi, PAK; 4 Internal Medicine, Wayne State University, Detroit, USA; 5 Radiology, Dow University of Health Sciences, Karachi, PAK

**Keywords:** contrast-induced nephropathy, st-segment elevation myocardial infarction, primary percutaneous coronary intervention, acute kidney injury

## Abstract

Background

Contrast-induced nephropathy (CIN) after primary percutaneous coronary intervention (PCI) is associated with increased mortality and morbidity. The aim of this study is to determine the frequency of CIN after primary PCI and its association with risk factors in patients with ST-segment elevation myocardial infarction (STEMI) at a tertiary care cardiac center in Pakistan.

Methodology

In this observational study, we included 282 patients who presented with STEMI and underwent primary PCI at the National Institute of Cardiovascular Disease, Karachi, Pakistan, from October 2017 to April 2018. The serum creatinine (mg/dL) levels were obtained at baseline and 48 to 72 hours after the primary PCI procedure, and patients with a 25% increase or ≥ 0.5 mg/dL rise in post-procedure creatinine level (after 48 to 72 hour) were categorized for CIN.

Results

Out of a total sample of 282 patients, 68.4% (193) were males, and the mean age was 56.4 ± 9.1 years. A majority of the patients, 78.7% (222), were hypertensive and 34% (96) were diabetic. The CIN was observed in 13.1% (37) of the patients, and increased risk of CIN was found to be associated with the presence of diabetes mellitus and increased (>200 mL) use of contrast during the procedure, with odds ratios of 2.3 (1.14-4.63) and 3.12 (1.36-7.17), respectively.

Conclusions

The CIN after PCI is a common complication associated with the presence of diabetes mellitus and the use of an increased amount of contrast during the procedure.

## Introduction

Primary percutaneous coronary intervention (PCI) is the recommended and preferred reperfusion strategy for the patients with ST-segment elevation myocardial infarction (STEMI). When performed within the 12-hour window of symptom onset, it is an effective treatment strategy associated with a significant reduction in mortality and morbidity as compared to thrombolysis [1]. A decrease in glomerular filtration rate after procedure due to injection of contrast media, known as contrast-induced nephropathy (CIN), remains a real risk and is associated with increased risk of mortality and morbidity, consequently leading to prolongation of hospital stay, increased utilization of resources, and increased healthcare cost [2,3]. In recent literature, the frequency of CIN after primary PCI is reported to range from 10.4% to 23.2% [2-8]. CIN is reported to be associated with increased in-hospital mortality rate after primary PCI, with around four to eightfold increased risk of in-hospital mortality [2,9].

Despite widespread use of contrast agents in intervention and radiographic studies, the pathophysiology of CIN is complex and not fully understood and involves various mechanisms, such as oxidative stress, vasoconstriction, medullary ischemia, and allergic reactions to contrast media [3,10]. Various patient- and procedure-related factors have been observed to be reasons that can the exaggerate acute kidney injury (AKI), such as preexisting chronic kidney diseases (CKDs), diabetes, hemodynamic alterations, and volume depletion due to cardiogenic shock or heart failure, complex interventional procedures, and use of increased amount of contrast during the procedure [2,11,12].

Potential preemptive strategies to minimize the CIN are risk stratification of high-risk individuals, optimization of volume status, monitoring of adequate hydrations and infusion of normal saline before and after the procedure, and use of minimum contrast amount during the procedure. Guidelines on various preventive nonpharmacological and pharmacologic agents are not clear, and there is poor concordance in the literature, and despite the varying degree of agreement, iso-osmolar contrast or nonionic low-osmolar contrast is the preferred media among the interventional cardiology [13,14].

CIN after primary PCI and its associated risk factors widely vary from study to study, and very limited data on our population are available regarding this important clinical condition; therefore, in this study our aim is to determine the frequency of CIN after primary PCI and its association with risk factors in patients with STEMI at a tertiary care cardiac center in Pakistan.

## Materials and methods

In this observational study, we included patients who presented to the emergency department with STEMI within 12 hours of the onset of symptoms and received primary PCI for revascularization at the National Institute of Cardiovascular Disease, Karachi, Pakistan, during October 2017 to April 2018.

The consented patients who fulfilled the inclusion criteria of the study were included consecutively. The Inclusion criteria were either gender, between 30 and 70 years of age, and normal serum creatinine level (<1.2 mg/dL) at baseline. Hemodynamically unstable patients, such as patients in shock or Killip class IV or those with pre-existing CKD or end-stage renal disease were excluded from the study.

Baseline demographic characteristics, such as gender and age (years), and clinical characteristics, such as history of diabetes, hypertension, and smoking, were recorded for all the patients. Diagnosis of STEMI was made based on history and electrocardiographic (ECG) changes at presentation, such as history of typical chest pain lasting for more than half an hour and supported by the baseline ECG findings of ST-segment elevation. Patients with a history of taking anti-hypertensive and anti-diabetic medication for at least six months were categorized as hypertensive and diabetic, respectively. Patients currently smoking or with a history of smoking a pack per day for at least one year were categorized as smokers.

The serum creatinine (mg/dL) levels were obtained at baseline and 48 to 72 hours after the primary PCI procedure, and patients with a 25% increase or ≥0.5 mg/dL rise in post-procedure creatinine level (after 48 to 72 hours) were categorized for CIN. The primary PCI procedures were performed by the consultant cardiologists, and the amount of contrast (mL) used during the procedure was also recorded.

Appropriate, frequency (percentages) or quantitative summary measures, such as mean ± standard deviation and minimum and maximum, were reported for the qualitative and quantitative characteristics of the patients. Stratifications were made by demographic characteristics (such as gender and age), co-morbidities (such as hypertension, diabetes, and smoking), and procedural characteristics (such as among of contrast used during the procedure), and odds ratios (ORs) along with 95% confidence interval (CI) were computed and the chi-square test was applied. Data analysis was performed with the help of IBM SPSS Version 19 (IBM Corp., Armonk, USA), and the level of significance was set at 5%.

## Results

In the study sample of 282 patients, 68.4% (193) were male patients, and the mean age of the study sample was 56.4 ± 9.1 years. Hypertension was the most common co-morbid condition, which was observed in 78.7% (222) patients. The mean serum creatinine level increased from 0.77 ± 0.16 mg/dL at baseline to 1.06 ± 0.19 mg/dL, p < 0.001, after 48 to 72 hours of the procedure. The CIN was observed in 13.1% (37) patients. The demographic and clinical characteristics of the patients included in this study are reported in Table 1.

**Table 1:** Demographic and clinical characteristics of the patients included in this study SD, standard deviation; CIN, contrast-induced nephropathy

Characteristics	Total
Total (N)	282
Gender	
Male	68.4% (193)
Female	31.6% (89)
Age (years)	56.4 ± 9.1
30 to 55 years	42.6% (120)
56 to 70 years	57.4% (162)
Co-morbid conditions	
Hypertension	78.7% (222)
Diabetes mellitus	34% (96)
Smoking	27.7% (78)
Baseline serum creatinine level (mg/dL)	
Range (minimum to maximum)	0.5 to 1.1
Mean ± SD	0.77 ± 0.16
Serum creatinine level (mg/dL) after 48 to 72 hours	
Range (minimum to maximum)	0.8 to 1.8
Mean ± SD	1.06 ± 0.19
Contrast amount used (mL)	
Range (minimum to maximum)	50 to 250
Mean ± SD	158.97 ± 43.49
≤200 mL	87.2% (246)
>200 mL	12.8% (36)
CIN	
Yes	13.1% (37)
No	86.9% (245)

In this study, we observed no statistically significant association between the CIN, and gender, age, presence of hypertension, and smoking status of the patients. However, increased risk of CIN was found to be associated with the presence of diabetes mellitus and increased (>200 mL) use of contrast during the procedure with ORs of 2.3 (1.14-4.63) and 3.12 (1.36-7.17), respectively. Rate of CIN by various patient demographic and clinical characteristics and associated ORs are presented in Table 2.

**Table 2:** Rate of contrast-induced nephropathy (CIN) by various patient demographic and clinical characteristics CIN, contrast-induced nephropathy; OR, odds ratio; CI, confidence interval **Benchmark; *Significant at 5%

Characteristics	Base (N)	CIN rate, % (n)	OR (95% CI)	p-Value
Total	282	13.1% (37)	-	-
Gender				
Male	193	15.5% (30)	2.16 (0.91-5.12)	0.076
Female**	89	7.9% (7)		
Age (years)				
30 to 55 years	120	12.5% (15)	0.91 (0.45-1.84)	0.791
56 to 70 years**	162	13.6% (22)		
Hypertension				
Hypertensive	222	14.4% (32)	1.85 (0.69-4.98)	0.216
Non-hypertensive**	60	8.3% (5)		
Diabetes mellitus				
Diabetic	96	19.8% (19)	2.3 (1.14-4.63)	0.017*
Non-diabetic**	186	9.7% (18)		
Smoking				
Smokers	78	12.8% (10)	0.96 (0.44-2.1)	0.926
Non-smokers**	204	13.2% (27)		
Contrast amount used (mL)				
>200 mL	36	27.8% (10)	3.12 (1.36-7.17)	0.005*
≤200 mL**	246	11% (27)		

## Discussion

CIN after primary PCI is an important complication reported to be associated with increased risk of in-hospital mortality, prolonged hospitalization, and post-procedure complications such as need of ventilator and major bleeding events [2]. In our study, 13.1% of the patients developed CIN after primary PCI, which was slightly higher than the reported incidence of 12.4% in the study by Batra et al. [2] and 10.2% in the study by Ullah et al. [15] for our population. These findings are aligned with the recent international studies, which reported CIN after primary PCI ranging from 10.4% to 23.2% [2-8]. A study based on NCDR CathPCI Registry by Tsai et al. [16] reported acute kidney injury in 7.1% of the patients after PCIs.

In our study, we observed that increased risk of CIN is associated with patients and procedure-related characteristics such as diabetes and increased amount of contrast used during procedure. A positive relationship between increased incidence of CIN and increased amount of contrast agent during procedure has been documented by various past studies [17,18]. Batra et al. [2] and Tsai et al. [16] had same observations regarding the amount of contrast and diabetes mellitus. In their observations, other patient-related characteristics such as female gender, CKD, hypertension, dyslipidemia, history of anemia, and congestive heart failure were significantly associated with the development of CIN after primary PCI. Also, patients who developed CIN had longer hospital stays, significantly higher in-hospital mortality rate, and increased post-procedure complications such as need of ventilator and bleeding [2,6,16]. It has been suggested that increased baseline serum creatinine can be taken as a surrogate marker of extensive and severe atherosclerosis, circulatory instability, and major adverse cardiovascular event [19].

Considering the implications of this complication in the current era of intervention, it is important to adopt preemptive preventive strategies, pharmacological or nonpharmacological, in these patients. In a multicenter clinical trial, Chong et al. [20] reported that 12-hour sustained sodium chloride pre-hydration regimen may prevent CIN. Jo et al. [21] in their study assessed high-dose atorvastatin for the prevention of CIN, but the evidence was not supportive of the role of high-dose atorvastatin in preventing CIN. Nawa et al. [22] reported that for patients with poor renal function, continuous intravenous infusion of nicorandil for 4 hours before and 24 hours after procedure strongly prevent CIN after elective PCI. Similarly, Firouzi et al. [23] argued prophylactic use of intravenous magnesium sulfate for the prevention of CIN.

In clinical setting, early recognition of patients at increased risk of development of CIN is crucial, and various risk stratification modalities have been proposed in various studies. Mehran et al. [24] developed a simple score for the stratification of patients at high risk of CIN that has been validated and used by various studies ever since [25]. Kurtul et al. [26] tested neutrophil-to-lymphocyte ratio (NLR) for the risk stratification and reported that increased NLR is an independent predictor of CIN among patients who underwent PCI for non-NSTEMI. Kurtul and Duran [27] reported that the fragmented QRS complex is an independent predictor of CIN after primary PCI in patients with STEMI. Similarly, Goussot et al. [5] reported potential use of N-terminal fragment of pro B-type natriuretic peptide for the identification of at-risk patients, a good predictive value of CHA2DS2-VASC score for the identification of patients at higher risk of CIN after urgent PCI for acute coronary syndrome has been reported by Kurtul et al. [7], and Ozturk et al. [8] concluded that the logistic clinical syntax score can be used to improve the accuracy of risk stratification for the development of CIN.

## Conclusions

In conclusion, CIN after primary PCI is a common complication, and patients with diabetes mellitus are at an increased risk of development of CIN. Use of an increased amount of contrast during primary PCI procedure significantly increases the risk of post-procedure CIN. Early risk stratification and preemptive preventive measures are needed in clinical practice to minimize the clinical burden of CIN after primacy PCI.
